# Caspase recruitment domain (CARD) family (CARD9, CARD10, CARD11, CARD14 and CARD15) are increased during active inflammation in patients with inflammatory bowel disease

**DOI:** 10.1186/s12950-018-0189-4

**Published:** 2018-07-11

**Authors:** Jesús K. Yamamoto-Furusho, Gabriela Fonseca-Camarillo, Janette Furuzawa-Carballeda, Andrea Sarmiento-Aguilar, Rafael Barreto-Zuñiga, Braulio Martínez-Benitez, Montserrat A. Lara-Velazquez

**Affiliations:** 1 Department of Gastroenterology, Inflammatory Bowel Disease Clinic, Instituto Nacional de Ciencias Médicas y Nutrición Salvador Zubirán, Vasco de Quiroga 15, Colonia Sección XVI, Tlalpan, CP, 14000 Mexico City, Mexico; 2 Department of Immunology and Rheumatology, Instituto Nacional de Ciencias Médicas y Nutrición Salvador Zubirán, Mexico City, Mexico; 3 Department of Endoscopy, Instituto Nacional de Ciencias Médicas y Nutrición Salvador Zubirán, Mexico City, Mexico; 4 Department of Pathology, National Institute of Medical Science and Nutrition “Salvador Zubirán”, Mexico City, Mexico; 5 Facultad de Medicina, Universidad Nacional Autónoma de México, Av. Ciudad Universitaria 3000, C.P. Coyoacán, 04360 México City, Mexico

**Keywords:** Innate, Receptors, CARD, Ulcerative colitis, Gene, PCR

## Abstract

**Background:**

The CARD family plays an important role in innate immune response by the activation of NF-κB. The aim of this study was to determine the gene expression and to enumerate the protein-expressing cells of some members of the CARD family (CARD9, CARD10, CARD11, CARD14 and CARD15) in patients with IBD and normal controls without colonic inflammation.

**Methods:**

We included 48 UC patients, 10 Crohn’s disease (CD) patients and 18 non-inflamed controls. Gene expression was performed by RT-PCR and protein expression by immunohistochemistry. CARD-expressing cells were assessed by estimating the positively staining cells and reported as the percentage.

**Results:**

The CARD9 and CARD10 gene expression was significantly higher in UC groups compared with CD (*P <* 0.001). CARD11 had lower gene expression in UC than in CD patients (*P <* 0.001). CARD14 gene expression was higher in the group with active UC compared to non-inflamed controls (*P <* 0.001). The low expression of CARD14 gene was associated with a benign clinical course of UC, characterized by initial activity followed by long-term remission longer than 5 years (*P =* 0.01, OR = 0.07, 95%CI:0.007–0.70). CARD15 gene expression was lower in UC patients versus CD (*P =* 0.004). CARD9 protein expression was detected in inflammatory infiltrates; CARD14 in parenchymal cells, while CARD15 in inflammatory and parenchymal cells. CARD9−, CARD14− and CARD15 − expressing cells were significantly higher in patients with active UC versus non-inflamed controls (*P <* 0.05).

**Conclusion:**

The CARD family is involved in the inflammatory process and might be involved in the IBD pathophysiology.

## Background

Ulcerative Colitis (UC) is one of the two forms of Inflammatory Bowel Disease (IBD). Epidemiologic data from developing countries reveals that the incidence and prevalence of IBD are increasing in different regions around the world [1]. Family-based studies have identified a number of familial IBD loci using nonparametric linkage analyses, where IBD1 locus contains NOD2 gene [2, 3]. The NOD2 or CARD15, the first gene associated to Crohn’s disease (CD), is an intracellular receptor of the Nod Like Receptor (NLR) family that binds to muramyl-dipeptide (MDP), the minimal bioactive component of bacterial peptidoglycan [4], and through Nuclear Factor κB (NF-κB) pathway, it regulates the expression of tumor necrosis factor (TNF) and other pro-inflammatory cytokines [5].

The CARD-containing adaptors and sensors represent an important family of 15 molecules involved in innate host defense against gastrointestinal pathogens and in the regulation of inflammatory responses. CARD15 has been found to be a key orchestrator of the intestinal mucosal barrier homeostasis through the regulation of intestinal microbiota as well as innate and adaptive immune responses [6–8]. It belongs to CARD family, which also includes CARD9, CARD10, CARD11 and CARD14, among others.

The CARD9 is an adaptor molecule found mainly in lymphoid tissues, it plays a role in the innate immune response to fungi, bacteria, virus and mycobacteria, linking innate immunity with T-cell differentiation and type 17 T helper (Th17)-cell development [9–12]; it is known that the presence of certain genetic variants of CARD9 implies an increased risk of IBD [13]. It participates in the activation of NF-*κ*B under dectin-1 (C-type lectin domain family 7-member A, *CLEC7A*) stimulation and interactions between commensal fungi and the c-type lectin receptor dectin-1 influence colitis [11], which haplotype rs2078178–rs16910631 was found to be associated with CD and UC [13]. It physically associates with CARD15 in response to MDP and regulates the activation of the kinases p38 and JNK, required for the expression of inflammatory cytokines [12–14]. With the appearance of double-stranded DNA (dsDNA) in the cytoplasm, it forms part of the signaling complex dsDNA-Rad50-CARD9 and culminates with activation of the transcription factor NF-kB and the generation of pro-IL-1β [15]. CARD10, CARD11 and CARD14 are members of the membrane-associated guanylate kinase (MAGUK) superfamily, and function as molecular scaffolds by using multiple discreet protein interaction domains to cluster receptors and cytosolic signaling molecules at the cell membrane [13–15]. They contain three defining interaction domains: the PSD-95/Dlg/Z0–1 homologous (PDZ) domain, the Src- homology (SH3) domain and the guanylate kinase (GUK)-like domain. Their CARD domain, interacts specifically with the CARD of BCL-10, and under different stimuli, they activate NF-κB [16]. These three molecules have shown distinct tissue distributions but share 50–60% sequence identity in the CARD and coiled-coil domains, and 20–30% identity in the PDZ, SH3 and GUK domains [17–19]. CARD10 is expressed in a variety of adult tissues like heart, kidney, liver and multiple cancer cell lines including colorectal adenocarcinoma SW480 cells [20]. It interacts with Bcl10 and MALT1 in the platelet-activating factor (PAF)-induced inflammatory pathway in intestinal epithelial cells, and the pro-inflammatory effects of PAF play prominent roles in the pathogenesis of IBD [21]. CARD11 is expressed in the spleen, thymus and peripheral blood leukocytes, it is an important component of the antigen-induced NF-κB signaling pathway in T cells [16], and interestingly, certain genetic variants were associated with an incremented risk of UC [3]. CARD14 gene expression has been found in placenta, skin and mucosa, and it is the only member of the family so far shown to be expressed in different alternatively spliced isoforms [22–25]; its role in the context of IBD is unknown. The aim of this study was to determine the gene expression and to enumerate the protein-expressing cells of some members of the CARD family (CARD9, CARD10, CARD11, CARD14 and CARD15) in patients with IBD and normal controls without colonic inflammation.

## Methods

### Study subjects

A total of 48 patients with UC (26 patients with active UC and 22 patients with UC in remission), 10 patients with ileocolonic CD (1 patient with active CD and 9 patients with CD in remission) and 18 controls without colonic inflammation were included in the study. All colonic biopsies from patients with IBD and controls were taken from colon mucosa inflamed whole colon in UC and right colon in CD patients.

The diagnosis of UC was performed by the presence of the following criteria: presence of chronic diarrhea with blood in stools, macroscopic endoscopic appearance and confirmation by histopathology.

All IBD patients were included during the period from January 2014 to July 2015, belonging to the Inflammatory Bowel Disease Clinic at the National Institute of Medical Sciences and Nutrition “Salvador Zubirán”. Relevant clinical and demographic information from all patients were collected from interview and medical records (Table 1). Variables analyzed were age at diagnosis, gender, type of medical treatment (5-aminosalycilates, steroids, thiopurines, biologic therapy), disease extension or location according to Montreal Classification, the presence of extra-intestinal manifestations such as articular affection, ankylosing spondylitis, sacroiliitis, sclerosing cholangitis, pyoderma gangrenosum, erythema nodosum or uveitis and clinical course classified as: initially active and then prolonged remission (first episode with activity and then long-term remission for more than 5 years), intermittent activity (> 2 relapses per year) and chronic continual activity (persistent activity despite medical conventional therapy).

**Table 1:** Demographic, clinical and laboratory variables of patients with UC and controls

VARIABLES	ACTIVE UC (*n* = 26)	REMISSION UC (*n* = 22)	CROHN’S DISEASE (*n* = 10)	CONTROL GROUP (*n* = 18)
Age				
Mean ± SD (years)	37.7 ± 10.5	39.0 ± 13.7	45.2 ± 19.7	47.7 ± 11.4
Median	38	35	41	52
Range	20–57	20–66	21–85	24–60
Gender				
Female (%)	13 (50)	12 (55)	2 (20)	11 (61)
Male (%)	13 (50)	10 (45)	8 (80)	7 (39)
Clinical course				
Relapsing-remitting (%)	80.7	22.7	0	NA
Remission (%)	15.3	77.2	87.5	NA
Continuous activity (%)	3.8	0	12.5	NA
Extra intestinal manifestations (%)	64	27.2	33	NA
Disease duration				
Mean ± SD (years)	6.03 ± 4.96	11.59 ± 6.7	7.2 ± 8.8	NA
Median	4.5	11.5	5.5	NA
Range	0–19	1–30	0–27	NA
Treatment				
5-aminosalycilates (%)	38.5	54.5	50.0	NA
5-aminosalycilates + steroids (%)	61.5	45.5	50.0	NA
Steroid dependent (%)	3.8	0.0	12.5	NA
Steroid resistant (%)	23.0	18.2	12.5	NA
Histopathology severity				
Inactive disease (%)	0	100	11	NA
Mild (%)	28	0	89	NA
Moderate (%)	24	0	0	NA
Severe (%)	48	0	0	NA
Dysplasia (%)	0	0	0	NA
Laboratory values				
Hemoglobin (g/dL), Mean ± SD	12.9 ± 2.9*	14.3 ± 1.4	13.9 ± 2.1	14.8 ± 1.8
Median	13.6	14.6	14.4	14.9
Range	5.6–18.3	10.7–16.4	8.9–16.5	10.6–17.6
Mean corpuscular volume (fL), Mean ± SD	83.4 ± 8.1	88.5 ± 5.0	89.2 ± 7.3	90.2 ± 5.3
Median	85.2	89.0	91.4	91.1
Range	62.3–93.9	79.5–98.1	72.0–96.3	73.5–95.7
Mean corpuscular hemoglobin (pg/cell),				
Mean ± SD	27.5 ± 3.3	30.0 ± 2.2	30.2 ± 2.6	30. 7 ± 2.2
Median	28.5	30.4	30.4	31.2
Range	20.2–31.8	25.8–33.8	24.0–32.9	23.6–33.2
Leukocytes (10^3^/μL), Mean ± SD	8.3 ± 2.5	6.8 ± 2.7	6.6 ± 3.1	7.0 ± 2.3
Median	8.6	6.2	5.5	6.6
Range	3.7–13.9	4.6–17.4	4.2–14.7	3.4–13.1
Neutrophils (%), Mean ± SD	62.5 ± 12.2	63.2 ± 8.5	70.5 ± 10.4	58.0 ± 9.7
Median	63.2	61.6	70.3	59.8
Range	35.0–85.0	46.6–81.2	53.4–91.0	39.4–75.6
Lymphocytes (%), Mean ± SD	26.5 ± 9.9	27.4 ± 8.3	20.5 ± 8.9	30.6 ± 9.4
Median	26.4	27.6	18.3	27.5
Range	10.0–44.0	14.8–44.5	6.6–35.4	15.2–50.5
Monocytes (%), Mean ± SD Median	7.9 ± 2.6	7.1 ± 2.2	7.9 ± 3.5	7.4 ± 1.8
Median	7.8	6.8	7.5	7.3
Range	3.9–12.8	2.5–11.1	1.8–14.5	3.7–11.0
Platelets units 10^3^/μL), Mean ± SD	375.3 ± 142.0	238.0 ± 89.7	183.4 ± 58.1	212.2 ± 70.0
Median	371.0	229.0	187.0	207.0
Range	155.0–869.0	68.0–436.0	94.0–227.0	105.0–325.0
Erythrocyte Sedimentation Rate (mm/h),				
Mean ± SD	23.6 ± 18.8	13.1 ± 12.0	8.0 ± 6.9	3.5 ± 1.9
Median	17.5	11.0	4.0	3.0
Range	2.0–66.0	2.0–46.0	2.0 - 19.0	2.0–6.0
Environmental exposure				
Tobacco (%)	54.0	72.3	44.4	22.2
Alcohol (%)	35.0	31.8	22.2	16.6
Comorbidities				
Allergies (%)	3.8	9	0	16.6
Autoimmune diseases (%)	3.8	13.6	11.1	16.6
Medical history				
Hypertension (%)	0	4.5	0	22.2
Overweight/obesity (%)	0	0	0	5.5
Diabetes (%)	0	0	11.1	11.1
Dyslipidemia (%)	0	4.5	0	22.2
Gynecologic diseases (%)	3.8	18.1	0	16.6
Surgery history (%)	61.5	68.1	55.5	55.5

*CD* crohn’s disease, *UC* ulcerative colitis

Hemoglobin *p* = 0.018 (active ulcerative colitis vs. control group)

Mean corpuscular volume *p* = 0.008 (active ulcerative colitis vs. control group) and *p* = 0.048 (remission ulcerative colitis vs. control group) Neutrophils *p* < 0.001 (Crohn’s disease vs. control group)

Erythrocyte sedimentation rate *p* = 0.042 (active ulcerative colitis vs. control group) and *p* = 0.0498 (active ulcerative colitis vs. Crohn’s disease)

Non-parametrical tests were used such as U Mann Whitney test for numerical variables and Fisher Exact test for categorical variables

Colonoscopy was performed to calculate the Mayo Score Activity Index and Riley for endoscopy and histology activity, respectively [26–28]. The disease severity was evaluated by Harvey-Bradshaw Index for CD patients [29].

In IBD patients, the colonic biopsies were taken from the whole colon in UC patients and right colon in CD patients, especially in the most severe inflammation evaluated by colonoscopy.

The histological analysis of those patients with active UC showed that 28% of the patients had mild activity, 24% moderate and 48% with severe histological inflammation. The remaining 22 UC patients in remission were confirmed by histopathology. Regarding to the treatment, all patients were taking sulfasalazine or 5-aminosalicylic acid (5-ASA); 47.5% used systemic steroids; 30% were taking thiopurines and 2.5% received anti-TNF therapy.

The control group consisted of non-inflamed controls (no documented inflammatory disease) undergoing to colonoscopy for other reasons and approving by written informed consent the use of specific colonic biopsies for this study.

### Sample processing and gene expression analysis

All colonic biopsies were taken by colonoscopy and were immediately placed in RNA later (Ambion, Austin, TX, USA) and stored at − 70 °C until processing. Then total RNA was isolated using High Pure RNA Tissue (Roche Diagnostics, Mannheim, Germany), following the manufacturer’s guidelines. Two hundred nanograms of total RNA were reverse-transcribed into cDNA with random hexamer primers (Roche Diagnostics, Mannheim, Germany).

The PCR amplification of the above mentioned genes was carried out with 20 ng of cDNA, 200 nM forward and reverse primer, and Taqman Master Mix (Roche Diagnostics, Mannheim, Germany Roche Diagnostics, Mannheim, Germany) in a final volume of 10 μl. PCR reactions were run in a Light Cycler 2.0 (Roche Diagnostics, Mannheim, Germany) for 45 cycles, each cycle consist in denaturation for 15 s at 95°, primer annealing for 15 s at 55°, and extension for 30 s at 72 °C and cooling 30 s at 40 °C.

The CARD gene expression was measured by real-time polymerase chain reaction (RT-PCR). Reference gene GADPH transcripts were used for relative quantification and quality controls. For q-PCR assays quality control, determination of linearity and reproducibility was evaluated (VC < 10%). The mRNA relative quantification of target genes was conducted using the LightCycler software 4.1, according to the 2-delta-delta Ct method. Table 2 shows the details of the primers designs used for the RT-PCR.

**Table 2:** Primers designs used for Real Time Polymerase Chain Reaction (RT-PCR)

GENE	GENEBANK	OLIGO LEFT	OLIGO RIGHT	UNIVERSAL PROBE LIBRARY	AMPLICON SIZE
CARD9	NM_052814.3, NM_052813.4	ggagacgctcgtcctgag	aggtcctgggggagtgag	79	gagacgctcgtcctgagctccgacctggaagatggctcacccaggaggtcccaggagctctcactcccccaggacct
CARD10	NM_014550.3	actaccccgaacacttcacg	gtcatcaagaattgggtcagg	72	actaccccgaacacttcacgctgctcacgggccaggaacccgcccagcgctgctccatgatcctcgatgaggaggggcctgagggcctgacccaattcttgatgac
CARD11	NM_032415.4	cagaggagctgcgagacaa	ggtgcttgtacatttcacagtcc	1	cagaggagctgcgagacaagtacctggaggagaaggaggacctggagctcaagtgctcgaccctgggaaaggactgtgaaatgtacaagcacc
CARD14	NM_024110.3	gagctcctagacacggcaga	cgagacatcaagccttccag	71	gagctcctagacacggcagaccttccgcagctggaaagcagcctgcagccagtctcccctggaaggcttgatgtctc
CARD15	NM_022162.1	gtgtcctcctcggacattct	ggaccctgagaccagcag	36	gtgtcctcctcggacattctccgggttgtgaaatgtgctcgcaggaggcttttcaggcacagaggagccagctggtcgagctgctggtctcagggtcc
GAPDH	NM_002046.3	agccacatcgctcagaca	gcccaatacgaccaaatcc	60	agccacatcgctcagacaccatggggaaggtgaaggtcggagtcaacggatttggtcgtattgggc

CARD9 assay was designed to detect both transcript isoforms (Universal Probe Library)

### Double staining immunohistochemistry procedure

A total of 10 surgical specimens from patients with active severe UC refractory to conventional therapy were included for ex-vivo protein detection as well as 10 patients with definitive diagnosis of ileocolonic active CD were enrolled in the study. Ten controls were obtained from non-inflamed non-IBD intestinal tissue.

In order to determine the CARD9, CARD14 and CARD15-expressing cells, 4 μm thick sections of formalin–fixed paraffin embedded tissue were placed on positively charged slides. Sections were deparaffinized with xylene and rehydrated with a graded series of ethanol and water washes before staining.

Follow the standard dewaxing and rehydration, enzyme antigen retrieval was performed (Enzo Life Sciences, Inc., Farmingdale, NY, USA). Tissues were blocked with a peroxidase solution. Then non-specific background staining was avoided with the IHC background blocker (Enzo Life Sciences). To determine the subpopulation of CARD15+/CD14+ and CARD9+/CARD14 cells a simultaneous detection was performed (MultiView (mouse-HRP/rabbit-AP) Enzo Life Sciences). The procedure is a sequential double staining where the IHC universal negative control reagent, as negative control (Enzo Life Sciences) and the mouse monoclonal anti-CARD9 IgG2a antibody (Santa Cruz Biotechnology, Santa Cruz, CA, USA)/rabbit polyclonal anti-CARD14, and the mouse monoclonal anti-CARD15 IgG1 antibody/rabbit polyclonal anti-CARD14 (Santa Cruz Biotechnology) at 10 μg/mL were incubated during 30 min at room temperature. Slides were washed and then incubated with PolyView IHC reagent (mouse-HRP) and PolyView IHC reagent (rabbit-AP) for 20 min. Finally, antigens were visualized using horseradish peroxidase (HRP)/3, 3′-diaminobenzidine (DAB) and the second antigen with alkaline phosphatase (AP)/Permanent Red. Tissues were counterstained with Harris’ hematoxylin and mounted with permanent mounting medium. CARD9^+^-, CARD14^+^- and CARD15^+^-expressing cells were assessed by estimating the positively staining cells in three fields (× 320) and were reported as the percentage of immunoreactive cells of the inflammatory infiltrates located at mucosa, submucosa, muscular and serosa. Results are expressed as the mean ± standard error of the mean (SEM) of cells quantified by the program Image Pro Plus version 5.1.1.

### Ethical considerations

This study was performed according to the principles expressed in the Declaration of Helsinki. The study was approved by the Ethical and Research Committee from the Instituto Nacional de Ciencias Médicas y Nutrición, and a written informed consent was obtained in all subjects.

### Statistical analysis

We used descriptive statistical analysis for demographical variables. The Kruskall Wallis is a non-parametric test for analyzing the differences in the gene expression between all groups. For categorical variables was used the Fisher’s exact test for non-parametric variables. The Odds Ratio (OR) was used in order to determine association between gene expression and clinical outcomes. Immunohistochemistry analysis was done by using one-way analysis of variance on ranks by Holm-Sidak method and Dunn’s test for all pairwise multiple comparison procedures. The Sigma Stat 11.2 program (Aspire Software International, Leesburg, VA, USA) and SPSS version 17.0 statistical program were used for the analysis. A *P* value below of 0.05 was considered as significant.

## Results

### Demographic and clinical characteristics

All demographic, laboratory, and clinical data from patients and controls are shown in Table 1. Regarding to disease activity, 26 had active disease and 22 were in remission according to Mayo and Riley Scores. The extent of disease was evaluated by using total colonoscopy and biopsies were taken from all segments of colon. The Montreal classification was used to define the extent of UC.

### CARD9, CARD10 and CARD11 gene expression in colonic tissue of UC patients

**Fig. 1:**
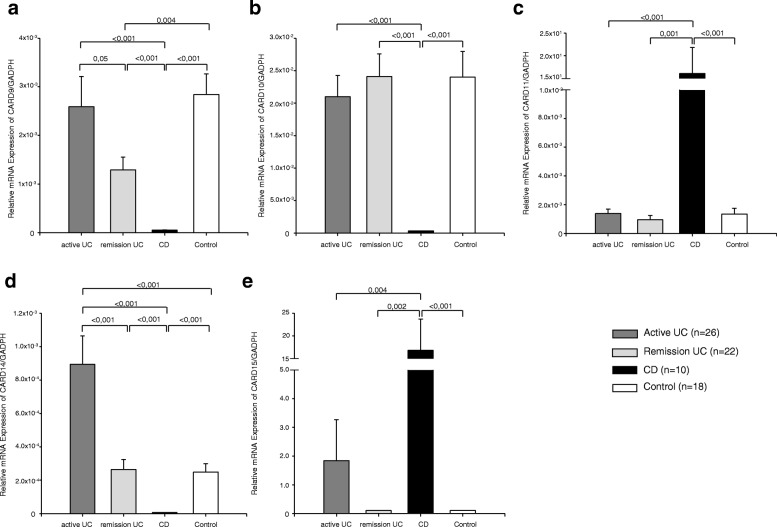
CARDs Gene Expression in Colonic Tissue of Ulcerative Colitis Patients. **a** CARD9; **b** CARD10; **c** CARD11; **d** CARD14; and **e** CARD15 relative gene expression quantified by RT-PCR. Bars show mean ± standard error of the mean of transcript levels in colonic mucosa from ulcerative colitis (UC) patients, active Crohn’s disease (CD) patients, and non-inflamed controls with GAPDH as housekeeping gene determined by 2-∆∆Ct. A non-parametric Kruskall Wallis test was used for analyze differences between groups

The CARD9 gene expression was higher in the active UC compared to the group with remission UC (*P =* 0.05) and CD group (*P <* 0.001). The CARD9 and CARD10 gene expression was higher in the remission group compared to inflamed CD (*P <* 0.001; Fig. 1a and b) and only CARD9 had lower levels than non-inflamed control group (*P =* 0.004; Fig. 1a). No significant associations were found between the CARD9 gene expression and clinical characteristics of IBD.

The CARD11 gene expression was lower in active and remission UC groups compared to CD inflamed group (*P ≤* 0.001). No statistically significant differences were found in CARD11 gene expression in UC versus non-inflamed control group (Fig. 1c).

### CARD14 gene expression was associated with a more benign clinical course

The CARD14 gene expression was higher and statistically significant in the group with active UC compared to inflamed CD and non-inflamed controls (*P* < 0.001; Fig. 1d). The low expression of CARD14 gene in remission UC patients was associated with a benign clinical course of the disease, characterized by initial activity followed by long-term remission longer than 5 years (*P =* 0.01, OR = 0.07, 95% CI: 0.007–0.70).

### CARD15 gene expression in colonic tissue of UC patients

CARD15 gene expression was significantly higher in patients with CD compared to active UC (*P =* 0.004). No statistical significant difference was found in the gene expression of CARD15 between UC in remission versus non-inflamed control group (Fig. 1e).

Finally, no significant associations were found between CARD 9, 10, 11 14 and 15 demographic and clinical characteristics in IBD patients including the medical therapy, phenotype and extent of disease.

### CARD9, CARD14 and CARD15 expressing cells in patients with UC and controls

**Fig. 2:**
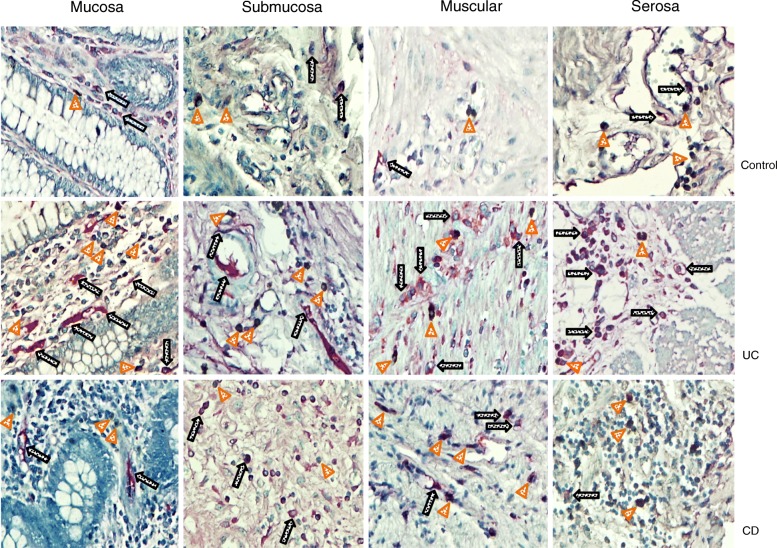
CARD9 and CARD14 protein expression in Colonic Tissue. Representative immunoperoxidase photomicrographs of non-inflamed control tissue, Ulcerative Colitis (UC) and Crohn’s disease (CD) colonic tissue. Orange arrowheads depict CARD9 immunoreactive cells in mucosa, submucosa muscular and serosa layer. Black arrows show CARD14-expressing cells. Original magnification was × 320. The statistical method used was one-way analysis of variance on ranks by Holm-Sidak method and Dunn’s test for all pairwise multiple comparison procedures

**Fig. 3:**
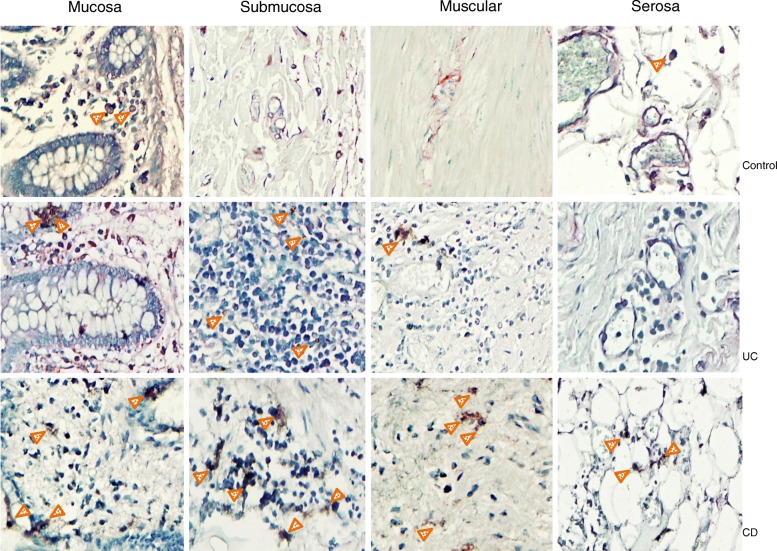
CARD15 protein expression in Colonic Tissue. Representative immunoperoxidase photomicrographs of non-inflamed control tissue, Ulcerative Colitis (UC) and Crohn’s disease (CD) colonic tissue. Orange arrowheads depict CARD15 immunoreactive cells in mucosa, submucosa muscular and serosa layer. Original magnification was × 320. The statistical method used was one-way analysis of variance on ranks by Holm-Sidak method and Dunn’s test for all pairwise multiple comparison procedures

**Fig. 4:**
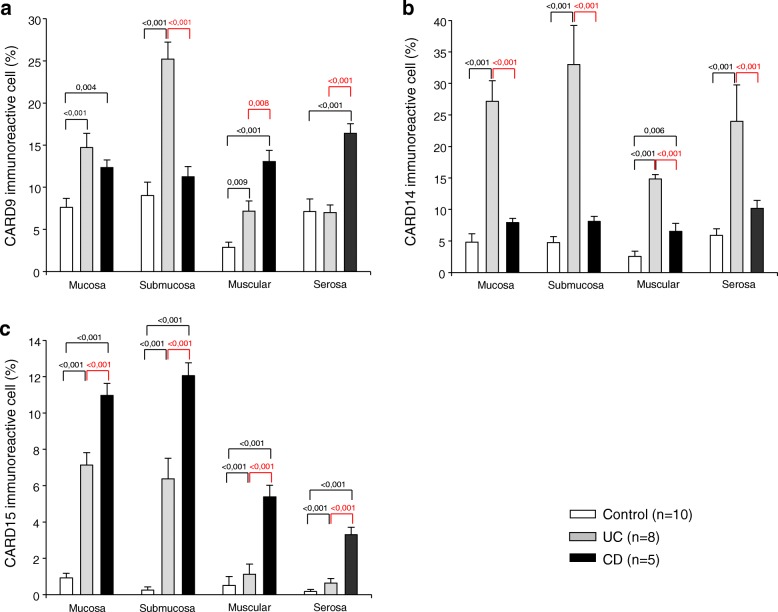
CARDs relative percentage expression in Colonic Tissue of IBD patients. Percentage of **a** CARD9, **b** CARD14, and **c** CARD15-expressing cells in active ulcerative colitis (UC), active Crohn’s disease (CD) and non-inflamed colonic tissue. The statistical method used was one-way analysis of variance on ranks by Holm-Sidak method and Dunn’s test for all pairwise multiple comparison procedures

Histochemical analysis showed that colonic tissue from non-inflamed controls had a baseline expression of CARD9, CARD14 and CARD15 less than 10% throughout the mucosa, submucosa, muscular and serosa layers (Figs. 2 and 3). Colonic tissue of UC patients had abundant inflammatory infiltrates, predominantly mononuclear cells, which extended from the serosa to mucosa, being more abundant in the epithelium, submucosa and serosa. CARD9 was expressed by inflammatory infiltrates (Fig. 2). The mucosa, submucosa and muscular from UC patients showed a statistically significant increase in CARD9^+^ cell percentage versus non-inflamed tissue control group. However, CARD9 levels in muscular and serosa were lower in active UC compared with active CD (Fig. 4a).

CARD14 was expressed by parenchymal and endothelial cells, (Fig. 2). UC patients had significantly higher levels of CARD14^+^ cells compared to non-inflamed and CD control groups (Fig. 4b).

CARD15 was expressed in inflammatory infiltrates and parenchymal cells (Fig. 3). CD patients had higher levels of CARD15-expressing cells throughout intestinal tissue layers compared to UC and non-inflamed control group. Nonetheless, CARD14 had significantly higher expression in tissue from UC patients versus non-inflamed control group (Fig. 4c).

## Discussion

CARDs family is composed by 15 molecules that are involved in innate host defense against gastrointestinal pathogens and in the regulation of inflammatory responses, suggesting that further insights into their physiological functions may yield new pharmacological strategies for treating intestinal inflammatory conditions [30].

The great interest to study the CARD family emerged from the impact that CARD15 has in the CD pathophysiology, regarding its implication in avoiding bacterial mucosal inflammation [7]. No previous studies have been evaluated in UC patients.

The findings of the present study showed that there is a differential gene and protein expression of some CARD family members due to the up-regulated expression of CARD9, CARD14 and CARD15, contrasting with the lack of significant expression in CARD 10 and 11 members in patients with UC.

Our findings suggest that CARD14 might be involved in the pathophysiology of UC, suggesting that CARD14 might play an important role concerning intestinal inflammation. Its relevance had previously been focused in patients with psoriasis, for instance, a Single Nucleotide Polymorphism (SNP) of this gene, named PSORS2 has been considered one of the major genetic risk factors for psoriasis. It has been proposed that *CARD14* mutations lead to enhanced NF-kB activation and up-regulation of a subset of psoriasis-associated genes in keratinocytes, which up-regulates an acute inflammatory response throughout excessive activation of NF-kB responsive genes and initiating the recruitment of the inflammatory infiltrate where CARD14 seems to be a regulator of skin inflammation [31]. Similar findings have been made regarding generalized pustular psoriasis [32] and pitiriasis rubra pilaris [33].

Our results are relevant regarding the study of CARD14 as part of the large list of molecules implicated in the puzzling study of IBD etiology, besides suggesting the importance of studying other members of the CARD family. Therefore, this is a fertile ground to other studies regarding the further events that this study was not possible to address.

For instance, in the context of skin immunology, CARD14 protein expression has been found to be reduced in the basal layer, and more diffusely up regulated in the suprabasal layers of the epidermis, unlike the normal CARD14 expression localized mainly in the basal and suprabasal layers of healthy skin epidermis [31].

On the other hand, CARD9 is an important adaptor of innate immune signaling pathways and has not been explored in UC patients. CARD9 is predominantly expressed in immune cells such as: myeloid dendritic cells, macrophages, T lymphocytes and B lymphocytes. Previously, Bertin J et al., showed that CARD9 expression in the small intestine and colon was low under normal conditions, but it is probably that increases during mucosal inflammation due to an influx of myeloid cells [17]. It has been demonstrated that CARD9 promotes recovery from colitis through activation of the IL-22 pathway. Moreover, *Card9*^−/−^ mice has an altered microbiota with increased load of gut-resident fungi, and reduced IL-22 production, and the transfer of microbiota from knockout mice to wild-type germ-free mice increases their susceptibility to colitis. In *Card9*^−/−^ administration of *Lactobacillus murinos, L. reuteri and L. taiwanensis* strains attenuates intestinal inflammation through catabolites generated by metabolism of tryptophan. These metabolites act as ligands for aryl hydrocarbon receptor (AHR) that can modulate IL-22 production. Interestingly, reduced AHR ligands production was also observed in patients with IBD, mainly in those with CARD9 risk alleles associated with IBD [34–36]. We found a protein overexpression of CARD9 in inflamed colonic tissue of UC groups compared to controls without inflammation. These results can be explained due to CARD9 is required for signaling from TLRs in dendritic cells and Triggering Receptor Expressed on Myeloid Cells (TREM) family of receptors in myeloid cells, since activation of nuclear factor-κB and/ or Mitogen-Activated Protein Kinases (MAPK), and induction of the proinflammatory cytokines TNF and IL-6. Probably, the increase of CARD9 observed in our patients could be a compensatory mechanism of inflammation due to dysbiosis. However, we cannot rule out the possible lack of functionality or poor function of the protein.

In genetic studies from other populations, the presence of certain genetic variants of CARD9 has shown to imply an increased risk of UC, and the haplotype rs2078178–rs16910631 was found to be associated with refractory UC [11, 13]. Zhernakova A et al., observed that the haplotype including the rs2287037*Grs1035130*G-rs2241116*C and rs6706002*A alleles occurred more frequently in IBD cases (26.1%) than in controls (21.4%) (*P* = 0.006) [11, 13].

In the same vein, CARD10 had not been explored in IBD patients. Our results showed that there is no significantly different gene expression of CARD10 in patients with active and remission UC compared to the non-inflamed control group.

Regarding the CARD11 gene expression no significant difference was found between UC patients and normal controls. The role of CARD11 mutations has been demonstrated in other types of diseases such as diffuse large B cell lymphoma [37], rheumatoid arthritis [38] and severe combined immunodeficiency [39]. In contrast, no previous studies have been performed in IBD patients.

We propose that further studies should be considered with a larger number of patients in order to find a statistically significant difference regarding CARD9 and CARD11 gene expression. This study entails the potential opportunity of finding one more possible explanation for the intense inflammation that characterizes IBD, waiting to be confirmed or rejected. The clinical relevance of these findings lies in the better understanding of UC pathophysiology.

Nevertheless, it has been suggested that not only the importance of the coiled-coil domains of CARD14, but also the ones of its related family members, such as CARD11, are important in regulating inflammation [40]. For this reason, attention needs to be further focused on the whole family role in intestinal mucosa immunity and the interaction between its members.

Although no risk alleles of NOD2/CARD15 have been associated in patients with UC, the NOD2/CARD15 gene expression was significantly higher in our cohort of patients with active UC compared to normal controls. No significant associations were found in the CARD15 gene expression regarding to clinical characteristics of UC. In this sense, Csillag et al., [41] previously demonstrated that gene expression profiling of mucosal biopsies from the descending colon of patients with CD could not be correlated with CARD15 status or with familial disposition for IBD. The over expression of CARD15 in CD immune cells was associated with expression of TNF and other pro-inflammatory cytokines, through NF-kB pathway [5].

Rosentiel P, et al. [42] demonstrated that TNF and IFN-γ up-regulate the expression of CARD15 gene in human intestinal epithelial cells and subsequently increased their LPS susceptibility, proposing that CARD15 could be part of the complex pathophysiology of barrier disruption, as it is observed in IBD. These results are of interest because they suggest an up-regulation of CARD15 gene expression in inflammatory conditions.

The findings related to gene and protein CARDs expression are based on the increased cellular infiltrates, activation of the cells and treatment, besides the mRNA presence not always is related to protein expression (correlation between the RNA and protein profile is ∼33 and 40%). It depends on half-life of different proteins (minutes to days) and/or posttranscriptional modification, RNA transport mechanisms, mRNA degradation (2–7 h), complex gene regulatory mechanisms, RNA processing, alternative splicing, RNA stability, and so forth [43].

Besides, samples used to determine mRNA expression are only from colonic mucosa, due to the fact that tissue was obtained by colonoscopy. Nonetheless for immunohistochemistry surgical specimens were employed.

This study has the strength of being the first depiction about the role of the CARD family in UC. This is the first time that other members of the family were studied and also the differential expression of CARD14 and CARD15 between UC and non-inflamed controls.

The role of CARD9 and CARD14 in the immunobiology of IBD is important in order to carry out studies in animal models by eliminating these receptors in specific cell lineages such as intestinal epithelial cells or cells from innate immunity.

The novel findings of the present study about gene expression of CARD9 and CARD14 can differently distinguish active and remission UC suggesting that blocking these receptors with an antagonist might be used as new therapeutic target in UC patients.

CARD14 gene expression was increased in patients with active UC, and its low gene expression was associated with a benign clinical course characterized by long-term remission, suggesting that an up-regulation is a defense mechanism in the colonic epithelia in response to decreased bacterial invasion and inflammatory activity.

## Conclusion

The CARD family might be involved in UC pathophysiology, due to the fact that CARD9, 14 and 15 gene expression was increased in patients with active UC.

The down-regulation of CARD14 was associated with a benign clinical course of UC characterized by long-term remission*.* Our results shed further light regarding the identification of CARDs in the pathophysiology of IBD.

## Abbreviations

CARD: Caspase Recruitment Domain
CD: Cronh’s Disease
IBD: Inflammatory bowel disease
RT-PCR: Real Time Polymerase Chain Reaction
UC: Ulcerative Colitis
